# Geographical Disparities in Pooled Stroke Incidence and Case Fatality in Mainland China, Hong Kong, and Macao: Protocol for a Systematic Review and Meta-analysis

**DOI:** 10.2196/32566

**Published:** 2022-01-18

**Authors:** Fan He, Irene Blackberry, Liqing Yao, Haiyan Xie, George Mnatzaganian

**Affiliations:** 1 John Richards Centre for Rural Ageing Research La Trobe Rural Health School La Trobe University Wodonga Australia; 2 Department of Rehabilitation Medicine The Second Affiliated Hospital of Kunming Medical University Kunming China; 3 Department of Health Care Peking Union Medical College Hospital Beijing China; 4 Rural Department of Community Health La Trobe Rural Health School La Trobe University Bendigo Australia; 5 The Peter Doherty Institute for Infection and Immunity Melbourne Australia

**Keywords:** case fatality, Hong Kong, incidence, Mainland China, Macao, meta-analysis, stroke, systematic review, stroke incidence, mortality rates, epidemiology

## Abstract

**Background:**

Geographical variations in stroke incidence and case fatality in China have been reported. Nonetheless, pooled estimates in major Chinese regions are unknown.

**Objective:**

This systematic review and meta-analysis aims to investigate pooled estimates of incidence and short-term case fatality of stroke in Mainland China, Hong Kong, and Macao.

**Methods:**

Longitudinal studies published in English and indexed in PubMed/MEDLINE, Embase, CINAHL, and Web of Science, or in Chinese and indexed in SinoMed and CQVIP will be targeted. Articles reporting on adults living in China who experience first-ever stroke or die within 1 year from newly onset stroke will be included. The 95% confidence intervals of the event will be estimated using the exact method based on the Poisson distribution. The log incidence rates together with their corresponding log standard errors will be meta-analyzed using DerSimonian and Laird random effects models. Pooled case fatality rates will also be estimated using a random effect model. Time trends in pooled age-standardized stroke incidence and case fatality will be estimated. The heterogeneity of the included studies will be measured using the I^2^ statistic and meta-regressions will be run to analyze the effect of reported covariates on found heterogeneity. Risk of bias will be examined using the Newcastle-Ottawa Scale. Publication bias will be tested using funnel plots and Egger tests. Sensitivity analysis will be run by risk of bias.

**Results:**

This study was funded and registered in 2020. The systematic searches, study selections, and quality assessments were completed in July 2021. Data extraction and analysis and manuscript writing are scheduled to be completed by December 2021.

**Conclusions:**

This will be the first study to provide regional differences in pooled estimates of stroke incidence with case fatality in Mainland China, Hong Kong, and Macao. This study will assist in addressing inequalities in stroke care across China.

**Trial Registration:**

PROSPERO International Prospective Register of Systematic Reviews CRD42020170724; https://www.crd.york.ac.uk/prospero/display_record.php?RecordID=170724

**International Registered Report Identifier (IRRID):**

PRR1-10.2196/32566

## Introduction

### Background

Stroke is the leading cause of death in China, contributing to one-third of global stroke-associated mortality [1-3]. Compared to high-income countries, including those in North America, Australia, and western and central Europe, China has lower stroke prevalence but higher incidence rate and mortality [2,4]. An ongoing stroke surveillance program covering 31 provinces in China reported an annual increase of 8.3% in the incidence of first-ever stroke in adults, from 189 per 100,000 population in 2002 to 379 per 100,000 population in 2013, with ischemic stroke and hemorrhagic stroke having incidences of 335 and 44 per 100,000 population, respectively, in 2013 [5]. By contrast, comparing 1990 with 2010, age-adjusted stroke mortality rates in China dropped from 167 to 126.9 per 100,000 population [6]. Reflecting stroke-associated productivity costs, patients bear the highest financial burden of stroke, with an estimated 33% of Chinese patients falling into poverty after experiencing stroke due to functional disability and health services costs [7].

Regional variations in the incidence of stroke and stroke-associated mortality across China are continuously reported [3,8-10], showing a north-south gradient; high and low incidence rates are reported in northeast and southeast Chinese coastal provinces, respectively [8,9], with rates being 2.4-fold higher in northeastern provinces [11]. A total of 9 provincial-level regions (Heilongjiang, Jilin, Liaoning, Inner Mongolia, Beijing, Hebei, Ningxia, Tibet, and Xinjiang) form a so-called stroke belt, covering north and west China, with stroke incidence being twice as high as that documented in provinces outside this belt [10]. Disparities in stroke mortality rates are similarly reported in urban and rural populations [12]. Significant geographical differences in mortality-to-incidence ratios have also been observed. The mortality-to-incidence ratio in southwest China is 0.68, which is considerably higher than the ratio of 0.42 found in east and south China [2].

### Gaps in the Literature

Considerable variations in the proportion of different pathologic types of stroke have been reported, with the proportion of intracerebral hemorrhage varying from 26.7% to 51.5% between Beijing and Changsha, which is a metropolitan city in central China [13]. Disproportionately higher rates of hemorrhagic stroke than ischemic stroke were reported in a 14-year study conducted in Changsha [14], which contradicts worldwide literature where ischemic stroke is reported at higher rates. By contrast, a nationwide study in 31 provinces showed that ischemic stroke was the major type of stroke in China, with the highest incidence rate being 166.9 per 100,000 population, constituting 70% of newly incident strokes [3]. Nonetheless, inconsistencies in stroke incidence continue to be reported across China [8], causing uncertainty regarding the real incidence rate among the Chinese population.

China is experiencing a decreasing trend in case fatality rates, with the overall rate of mortality per 100,000 population decreasing by 31% in urban or suburban regions and 11% in rural regions compared to 3 decades ago [15]. Although the proportion of patients with severe stroke admitted to best performing hospitals in China increased from 2007 to 2010, in-hospital case fatality following stroke hospitalizations dropped from 3.16% to 2.30% [16]. Underlying factors contributing to the downward trend of stroke case fatality in China may include improved treatments, broadened health care coverage, enhanced preventative campaigns, and public health literacy of stroke [15]. However, inconsistencies in stroke case fatality estimates have also been reported in China, ranging from 6.5% to 77.3% [8], which may have resulted from differences in inclusion and exclusion criteria used, resulting in samples that were not representative of the overall Chinese population diagnosed with acute stroke. Similarly, pooled estimates of the incidence of different types of stroke in China are currently unknown. Hence this systematic review (SR) and meta-analysis aims to estimate the pooled incidence and short-term case fatality of acute and nonrecurrent ischemic and hemorrhagic stroke in Mainland China, Hong Kong, and Macao. The evidence-based findings of the proposed study will inform policy making in stroke management and stroke prevention in China.

## Methods

### Study Registration

The protocol of this proposed study has been registered in the International Prospective Register of Systematic Reviews (PROSPERO): CRD42020170724.

### Criteria for Study Selection

#### Definition of Stroke

This study will use the World Health Organization (WHO) standard definition of stroke: “rapidly developed clinical signs of focal (or global) disturbance of cerebral function, lasting more than 24 hours or leading to death, with no apparent cause other than of vascular origin” [17]. Studies reporting the International Statistical Classification of Diseases and Related Health Problems (ICD) 10th version codes I60 to I64 and equivalent codes in earlier version will also be eligible for inclusion [18].

#### Inclusion and Exclusion Criteria

##### Inclusion Criteria

Studies that meet the following criteria could be included in the meta-analysis:

- Human adult populations (≥18 years old) experiencing an acute stroke or acute stroke-related death occurring within 1 year following diagnosis.
- Prospective or retrospective cohort studies reported in either English or Chinese.
- Study populations living in Mainland China, Hong Kong, or Macao.
- Sample size equal to or greater than 100 individuals. Studies reporting on less than 100 individuals will be regarded as case reports [19].

##### Exclusion Criteria

Studies that meet the following criteria could be excluded from the meta-analysis:

- Reporting on prevalent stroke or prevalent stroke-associated death. Admissions with acute stroke that occurred 48 hours after diagnosis will be considered as prevalent cases.
- Reporting on recurrent stroke or not clearly documenting past history of stroke.
- Reporting on transient ischemic attacks, silent cerebral infarcts, iatrogenic stroke, trauma-related or injury-related stroke, epidural hemorrhage, or central retinal artery occlusion.
- Case reports, case series, case-control studies, cross-sectional studies, studies with experimental or quasi-experimental designs, ecological studies, qualitative studies, abstracts without full text, comments, letters to the editor, newspaper articles or other non–peer reviewed grey literature, government reports, book chapters, reviews, and study protocols.
- Published before 1990 as before this year, computerized tomography (CT) and magnetic resonance imaging (MRI) were not widely used for stroke diagnosis in China [20].
- Data cannot be extracted.
- Denominators for estimating incidence and case fatality are not reported.
- No follow-up periods.

#### Other Considerations

Papers using the same data published in both English and Chinese will be included once. If multiple publications relate to the same study population, the study with the most complete data will be included.

### Search Strategy

#### Electronic Searches

Systematic searches will target the following electronic bibliographic databases: PubMed/MEDLINE, Embase, CINAHL, Web of Science (for studies published in English), SinoMed and CQVIP (for studies published in Chinese). Subject headings, MeSH terms, keywords of incidence, and country will be searched in all fields, including the title, abstract, and full text. The searches of stroke and mortality will be limited to the title and abstract. The dates and numbers of matched studies of searches of all databases will be recorded. The search strategies for Chinese and English publications will be similar (Multimedia Appendix 1 displays the terms and keywords used in the search strategy)

#### Hand Searches

SRs on the topic, detected in the searches, will be hand searched for potentially eligible articles which were not identified in the aforementioned searches.

### Data Collection

#### Selection of Studies

After removing duplicates, a team of researchers from La Trobe University, the Second Affiliated Hospital of Kunming Medical University, and Peking Union Medical College Hospital will follow the same screening process. Following the first screening, based on title and abstract, the full text of potentially eligible studies will be further screened. A total of 20% of the included studies in each step will be randomly and independently redone. Study authors will be contacted if additional details are needed for determining eligibility. All screened, excluded, and finally included articles will be reported in a Preferred Reporting Items for Systematic Reviews and Meta-Analyses (PRISMA) chart. This protocol was prepared in accordance with the PRISMA protocol (PRISMA-P) checklist.

#### Study Outcomes

Incidence of stroke will be measured as the number of new cases of stroke per 100,000 person-years. Short-term (1 month, 3 months, 6 months, and 1 year) case fatality will be reported as the ratio of the number of fatal cases to the total number of acute stroke cases. The Chinese standard populations over 3 decades, between 1990 and 2020, will be used to compute the age-standardized incidence and fatality rates for each historical period, when there is extractable data to do so.

#### Data Extraction and Management

Data will be extracted on the following covariates: date of publication, reporting language, authorship, regions under study, research design, sample size, time frame of the study, research setting (ie, community or hospital), information on the participants (age, sex, and smoking status), classification of stroke subtypes (ischemic or hemorrhagic), standard of diagnosis, and severity of stroke.

### Risk of Bias Assessment

The quality and risk of bias (ROB) of all finally included articles will be examined using the Newcastle-Ottawa Scale [21]. This scale examines the ROB of observational studies using 8 items, grouped into 3 elements: the sample selection, the comparability of study groups, and outcome ascertainment. One asterisk is awarded to the study for each item within the elements of sample selection and group comparability, and 2 asterisks are assigned to comparability, with 9 asterisks indicating the highest quality. In this SR, the quality of each included study will be further catergorized as good, moderate, or poor according to the thresholds for transforming the Newcastle-Ottawa Scale into the Agency for Healthcare Research and Quality standards [22].

### Disagreement Management

The coauthors will examine data extraction and quality evaluation independently. The level of agreement among reviewers will be estimated using the Cohen kappa coefficient. All inconsistencies in the screening process, assessment of the ROB, and data extraction will be discussed to make the final decision on study inclusion or exclusion, and quality assessment.

### Data Analysis

The exact method, based on the Poisson distribution, will be used to calculate 95% confidence intervals and standard errors of first-ever stroke. The incidence rates of disease will be expressed as Poisson means, estimated as the observed number of stroke events and probabilities relevant to the chosen confidence level, divided by time at risk. The log incidence rates together with their corresponding log standard errors, stratified by the 7 major Chinese geographical regions and 4 economic regions if there are enough data, will be meta-analyzed using DerSimonian and Laird random effects models [23]. Fixed effects models will also be considered. The pooled estimates will be age-standardized using the direct method, with the Chinese general population considered as standard. Case fatalities by region will also be meta-analyzed using random effect models. Region-specific analyses of pooled estimates of linear trends over time will be assessed using a chi-square test. The I^2^ statistic will be used to assess the heterogeneity of included studies [24]. The effect of reported covariates (ie, sample size; research setting, whether community or hospital; severity of stroke; and age, sex, and smoking status of the participants) on the heterogeneity in estimates of incidence and mortality among different studies will be evaluated using meta-regression. Subgroup analyses will be conducted by region. Funnel plots and Egger tests will be used for evaluating publication biases.

All analyses will be done separately for ischemic, hemorrhagic, and total stroke. Sensitivity analyses will be run by study setting (ie, community or hospital), publication language, and ROB.

All analyses will be conducted using Stata/SE, version 15.1 (StataCorp LLC). Stata’s metan command will be used to run the meta-analysis.

## Results

The systematic searches, study selections, and quality assessments were completed in July 2021. Data extraction and analysis and manuscript writing are scheduled to be completed before December 2021.

## Discussion

Pooled acute stroke incidence and case fatality rates in China are unknown. Reported estimates in the same or different regions vary considerably, making it hard to know the true incidence and fatality rates in the general Chinese population. Inconsistencies in these estimates may arise for various reasons [2,8]. Studies reporting incidence of stroke often use different diagnostic criteria, including definitions based on WHO criteria [25], Chinese National Stroke Conference criteria [8], or those based on ICD 9th or 10th codes [26]. Different methods of case ascertainment may also be used, which can lead to variations in incidence and case fatality rates. Examples of case ascertainment include neuroimaging (CT or MRI), medical records, self-reports, death registry data, and insurance claim records. Furthermore, different reference populations for age-standardization are often used, for instance, the world standard population [8], the US population [8], or the Chinese population [27]. Discrepancies in case fatality estimates may also be caused by the inclusion of different etiological types of stroke, recurrent or first-ever stroke, different onset ages, different levels of severity, and different selection criteria of included patients. Moreover, in China, disease incidence is often measured using household survey methods that lack follow-up periods [3,28,29]. Such commonplace cross-sectional surveys, which are more suitable to measure prevalence and burden of disease rather than incidence of disease, can produce potentially biased estimates because of nonresponse bias, reporting bias, and sampling errors due to the exclusion of certain groups from the sampling frames [30].

In this study, several measures will be undertaken to minimize potential biases in using published incidence and case fatality estimates. The SR will abide by strict inclusion and exclusion criteria to ensure that the included cases are representative of the Chinese general population. Acute stroke will be restricted to first-ever cases, with studies enrolling cases after 48 hours from diagnosis being excluded. Only studies that report a clear follow-up time will be considered. Cross-sectional surveys over a period of time without prospective or retrospective follow-up of participants will be excluded. Age standardization will be based on the Chinese general population. Furthermore, the choice of 1990 as the earliest publication date will ensure the validity of case ascertainment as, after that year, CT and MRI became more widely used in China [20].

This SR has strengths, but it also has some potential limitations. Metropolitan and economically developed regions in the northern and eastern coastal regions are likely to be overrepresented as research data are less available from less developed regions in west China [10]. Although this SR aims to remove all duplicate studies, it is possible that studies in similar locations may be reporting data on overlapping samples. If the authors did not explicitly describe their included samples, the inclusion of overlapping samples is likely. This SR will not investigate the subtypes of ischemic and hemorrhagic stroke, although the incidence, case fatality, and prevention and management strategies of their subtypes vary significantly. This study will investigate all-cause mortality following first-ever stroke instead of stroke-related death; however, our SR will only focus on 1-year all-cause mortality, which is more likely to have been caused by the stroke event.

Striking inequalities are reported in both the availability and quality of health care in China. Disparities in regional Healthcare Access and Quality scores are consistent with differences in the number of medical doctors per 1000 population and the proportion of designated stroke centers among secondary and tertiary hospitals in different provinces in China [2]. Investigating the stark differences in stroke epidemiology in different regions of China will be an important step in understanding these geographical differences. Our SR findings have the potential to better inform and influence clinical practice and policy making to address the regional inequalities in stroke-associated health outcomes in China.

### Funding

This study was supported by the 2020 China Studies Seed-funding Research Grant Scheme from La Trobe University, Australia.

## Appendix

Multimedia Appendix 1 Terms and keywords used in search strategy.

## Abbreviations

CT: computerized tomography
ICD: International Statistical Classification of Diseases and Related Health Problems
MRI: magnetic resonance imaging
PRISMA: Preferred Reporting Items for Systematic Reviews and Meta-Analyses
PRISMA-P: PRISMA protocol
PROSPERO: International Prospective Register of Systematic Reviews
ROB: risk of bias
SR: systematic review
WHO: World Health Organization
